# Umbilical Cord Mesenchymal Stem Cell-Derived Exosome-Mediated Regeneration in Hypertrophic Burn Scars Treated With Dual-Wavelength Laser: A Case Report

**DOI:** 10.7759/cureus.107906

**Published:** 2026-04-28

**Authors:** Ruri Pamela, Chi-Chih Kang, Lenny Lien, Grace Lee, Kyuho Yi

**Affiliations:** 1 Dermatology, Venereology and Aesthetic General Soedirman National Defense Central Hospital, Jakarta, IDN; 2 Project Management, BIONET Therapeutics, Taipei, TWN; 3 Medicine, BIONET Therapeutics, Taipei, TWN; 4 Executive Office, BIONET Therapeutics, Taipei, TWN; 5 Anatomy, Yonsei University, Seoul, KOR

**Keywords:** exosome, hypertrophic burn scar, mesenchymal stem cell, microneedling, non-ablative fractional laser, tissue regeneration, umbilical cord

## Abstract

Hypertrophic scarring following second-degree thermal injuries presents significant functional and cosmetic burdens, often characterized by dysregulated collagen deposition and persistent inflammation. Emerging multimodal treatment protocols involving the combination of regenerative medicine and non-ablative laser treatments offer novel scar management potential. We report the case of a 46-year-old woman presenting with a symptomatic hypertrophic scar resulting from a thermal injury sustained one month prior. The patient underwent a combined treatment protocol consisting of two sessions of fractional laser treatment (1550 + 577 nm), immediately followed by the application of umbilical cord mesenchymal stem cell (UCMSC)-derived exosomes via microneedling. Following two treatment courses, the patient demonstrated significant clinical improvement, characterized by a marked reduction in pigmentation, decreased erythema, and improved tissue pliability. This observed treatment efficacy is likely attributed to the synergistic effect of the ability of fractional lasers to create photothermolysis for remodeling fibrotic tissues, and the regenerative ability of UCMSC-exosomes to mitigate local inflammation and promote a controlled wound healing process. This case highlights the treatment potential of a multimodal protocol for managing hypertrophic burn scars. Further prospective, controlled studies are necessary to determine if exosome integration offers a definitive clinical advantage over energy-based monotherapies and to establish standardized treatment parameters.

## Introduction

Burn injuries frequently result in hypertrophic scarring, contractures, and pigmentary alterations that pose significant physical and psychological challenges for patients [1]. Unlike keloid scars, which grow beyond the original wound boundary and rarely regress, hypertrophic scars remain confined to the wound margins and may partially resolve over time, yet often persist with significant symptoms. Post-burn hypertrophic scars are associated with persistent inflammation, dysregulated fibroblast activity, excessive collagen deposition, abnormal angiogenesis, and sensory symptoms such as pruritus and pain, necessitating complex multimodal management strategies [1,2]. These pathological features are particularly impactful in facial and periocular regions, where even subtle scarring changes can significantly affect both function and aesthetic appearance.

Current standards of care for scar management primarily rely on physical modalities. Since the updated international clinical recommendations on scar management were published in 2014, the application of laser therapy has been at the forefront of the treatment, especially ablative and non-ablative fractional lasers [3-5]. The use of non-ablative fractional laser at 1550 nm creates microthermal zones (controlled microscopic columns of thermal injury that stimulate collagen remodeling and skin repair), resulting in dermal remodeling to improve dyschromia, hypertrophy, and skin texture [6]. However, physical resurfacing via the laser treatment alone has limitations. Aggressive laser settings, required for deep scars from second- or third-degree burn injuries, increase the risk of thermal injury to the epidermis, and post-inflammatory hyperpigmentation (PIH - darkening of the skin as a result of excess melanin deposition following cutaneous inflammation or injury), particularly in patients with higher Fitzpatrick skin types [7,8].

With a deeper molecular understanding of scar formation and to potentially mitigate the risk of PIH, the integration of regenerative biologics with laser therapy offers a promising new frontier in dermatological therapy. Platelet-rich plasma (PRP) [9] has been demonstrated to be an effective adjunct to laser therapy for hypertrophic burn scar management. However, autologous PRP quality is often limited by patient-dependent variability and logistical requirements of blood draws and processing. In contrast, allogeneic exosomes, especially mesenchymal stem cell-derived exosomes (MSC-exosomes), represent a cell-free, highly stable product for regenerative therapy. MSC-exosomes function in immunomodulation and extracellular matrix remodeling, prevent fibrotic scar formation, and promote angiogenesis to enhance wound healing [10]. Recent evidence suggests that exosome-augmented microneedling and CO2 laser therapies enhance skin rejuvenation, improving skin texture, hydration, elasticity, and pigmentation, without serious adverse events reported [11]. Additionally, clinical evaluations of combined exosome and microneedling treatments demonstrated a 4- to 6-point reduction in the Post-inflammatory Hyperpigmentation Area and Severity Index (PIHASI) in PIH patients, underscoring the efficacy of this multimodal approach to reduce the risk of PIH [12]. Combined treatment of human adipose tissue stem cell-derived exosomes and fractional laser showed reduced erythema and post-treatment downtime [13]. Building upon these successes, here, we present a second-degree thermal burn case managed with the multimodal treatment protocol at the General Soedirman National Defense Central Hospital, Jakarta, Indonesia. To our knowledge, this is the first reported case utilizing a dual-wavelength fractional laser protocol (1550 nm + 577 nm) in combination with UCMSC-derived exosomes delivered via microneedling for the management of hypertrophic burn scars in a patient with Fitzpatrick skin type IV. While prior studies have explored exosome-augmented laser therapies using adipose-derived stem cell exosomes for acne scars and skin rejuvenation, the application of this specific multimodal combination to post-burn hypertrophic scars - particularly in a cosmetically sensitive periocular region - represents a clinically distinct and underexplored indication. This case report documents the successful application of combining two fractional lasers and the umbilical cord mesenchymal stem cell-derived exosomes (UCMSC-exosomes) to improve the treatment of hypertrophic burn scar.

## Case presentation

**Figure 1:**
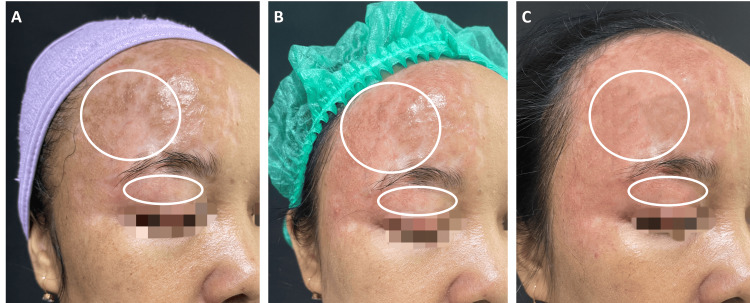
Sequential improvement of a second-degree thermal burn scar via combined fractional laser and microneedling-assisted UCMSC-exosome therapy. A. Initial presentation of the burn scar on the face caused by a hot oil splash one month prior. The right forehead (large white circle) exhibited localized hyperpigmentation and surface irregularity compared to the adjacent skin. The upper eyelid (small white oval) demonstrated pronounced asymmetric erythema, with greater involvement at the right side. B. At the one-month follow-up after the first treatment session, the right forehead (large white circle) revealed reduced hyperpigmentation whereas the upper eyelid (small white oval) showed less erythema and reduced superficial vascularization. C. At the one-month follow-up after the second session, both the right forehead (large white circle) and the upper eyelid (small white oval) demonstrated significant improvement, characterized by a smoothed burn scar texture and a more uniform skin color. The clinical photographs presented here were obtained from the patient described in the case presentation. Formal written informed consent was obtained from the patient for the publication of these clinical details in accordance with the journal’s ethical guidelines.

A 46-year-old woman presented with a symptomatic post-burn scar on the right forehead involving a portion of the upper eyelid (Figure 1A). The injury occurred approximately one month prior, due to a second-degree thermal burn from hot oil splashing onto the face while cooking. At the time of consultation, the patient reported persistent pruritus, a burning sensation, and intermittent pain described as a pulling or tight sensation at the scar site. The initial treatment included only normal saline-soaked gauze, oral antibiotics, and systemic analgesics for the wound management, insufficient for improving the symptoms of this patient. The patient also expressed aesthetic concerns, particularly due to the facial location of the lesion. On physical examination, the scar appeared hypertrophic, erythematous, irregular in texture, and with uneven pigmentation. No signs of active infection or ocular involvement were observed. Prior to confirming the diagnosis of hypertrophic scarring, differential diagnoses were considered, including keloid formation and persistent post-burn inflammation. Keloid was excluded based on the scar remaining confined within the original wound margins without lateral extension. Active infection and contact dermatitis were ruled out based on the absence of purulent discharge, vesiculation, or relevant exposure history.

Considering the symptomatic nature of the scar and its cosmetically sensitive location, a novel multimodal treatment approach involving fractional laser followed by microneedling-assisted exosome delivery was selected. A topical anesthetic cream (EMLA cream, AstraZeneca, Sweden) was applied prior to the procedure. The treatment area was subjected to a sequential dual-wavelength laser protocol using the Flexsys® platform (German Medical Engineering, Germany). First, dermal remodeling was initiated using a 1550 nm non-ablative fractional laser. Two passes were delivered at an energy of 30 mJ, with a fractional coverage of 8%, utilizing integrated air cooling to protect the epidermis. This was immediately followed by a 577 nm laser to target the vascular component of the scar. Two passes were delivered at a fluence of 20 to 25 J/cm², with a pulse duration of 20 ms, a fractional coverage of 30%, and the gel cooling setup. Immediately following the laser treatment, 1~2 mL of UCMSC-exosomes (Exocake, BIONET Therapeutics, Taiwan) were applied topically assisted with microneedling (REA-PEN, BeauMed, South Korea). The needle depth was 0.75 mm to enhance transdermal delivery. The exosome solution contained a concentration of 1.1 × 10^10 particles per vial. The treatment protocol consisted of two identical treatment sessions, spaced one month apart. Post-treatment care included the topical application of a soothing barrier-repair moisturizer to reduce erythema and support epidermal recovery. The patient was instructed on strict photo-protection using broad-spectrum sunscreen to prevent PIH.

At the one-month follow-up after the first session, the patient reported a significant reduction in pruritus, erythema, and pain. Clinically, the scar demonstrated visible improvement in texture and color uniformity (Figure 1B). At the one-month follow-up after the second session, the patient reported complete resolution of pruritus and pain, with patient-reported satisfaction consistent with the observed clinical improvement. Further improvement in skin smoothness and scar pliability was noted, with a more even surface texture and reduced erythema (Figure 1C).

Based on the Burn Scar Index at Vancouver Scar Scale (VSS), the scar in this patient shows a clear clinical improvement over time (Table 1). Initially, the scar demonstrated mild to moderate vascularity (pink-red discoloration), slight hyperpigmentation compared to surrounding skin, reduced pliability with a firm and shiny surface, and minimal scar height (<2 mm), resulting in an estimated total VSS score of approximately 7-9. Following treatment, the scar appears markedly improved, with vascularity returning close to normal, pigmentation becoming more uniform with adjacent skin, significantly increased pliability, and no detectable height, corresponding to a reduced VSS score of approximately 2-4 after two treatment sessions. This substantial decrease in VSS score indicates effective scar remodeling and restoration of skin characteristics toward normal, which is particularly relevant in facial scars and patients with skin of color.

**Table 1:** The VSS score of the patient at baseline and post-treatment based on the Burn Scar Index [14]

VSS Parameter	Baseline Assessment	Post-Treatment (2 Sessions)	Normal Skin Reference
Vascularity	Pink-red discoloration (Mild-Moderate) 1-2	Normal 1	0 (Normal)
Pigmentation	Slight hyperpigmentation 2-3	Uniform with adjacent skin 0-2	0 (Normal)
Pliability	Firm and shiny (Reduced) 3	Significantly increased 1	0 (Normal)
Height	Minimal (< 2 mm) 1	No detectable height 0	0 (Flat)
Total Score	7–9	2-4	0

No adverse events or complications were observed throughout the treatment period. No complication of PIH was observed in this patient, suggesting a potential anti-inflammatory protective effect of the exosome application.

## Discussion

The management of burn scars requires addressing two distinct pathological components, i.e., the structural dysregulation of collagen (fibrosis) and the biological environment of chronic inflammation. This case report suggests the potential efficacy of a combined treatment protocol for hypertrophic burn scars, utilizing a fractional laser to physically initiate scar tissue remodeling via photothermolysis, followed by the exosome treatment to biologically modulate inflammation and tissue regeneration. The primary distinction between our protocol and previous adipose-derived stem cell (ADSC) exosome studies lies in both the biological source and the laser system employed. UCMSC-derived exosomes offer superior batch-to-batch consistency and greater scalability as a standardized allogeneic product compared to adipose-derived preparations. Unlike prior studies that utilized a single-wavelength ablative CO₂ fractional laser targeting acne scars, our protocol employs a non-ablative dual-wavelength approach - combining 1550 nm for dermal remodeling with 577 nm specifically to address the vascular hyperreactivity component unique to hypertrophic burn scars. Exosome delivery was further optimized via microneedling at 0.75 mm depth to facilitate active transdermal penetration. Additionally, we provide preliminary proteomic and miRNA microarray data identifying key bioactive molecules, including NT5E/CD73, miR-21, FGF, HGF, TGF-β, and Decorin, offering a mechanistic basis for the observed outcomes that have not been reported in prior adipose-derived exosome studies.

**Figure 2:**
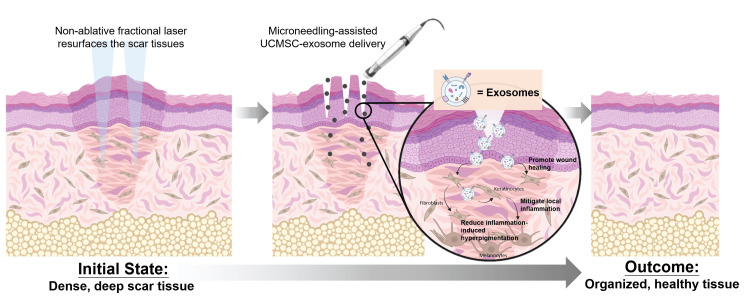
The schematic illustration demonstrates the multimodal treatment protocol for post-burn hypertrophic scar management. The treatment protocol includes physical scar resurfacing by the non-ablative fractional lasers, followed by skin tissue regeneration by microneedling-assisted exosome delivery. The UCMSC-exosomes transferred bioactive molecules to the keratinocytes or fibroblasts, promoting wound healing and mitigating local inflammation. The decreased pro-inflammatory cytokines reduce inflammation-induced hyperpigmentation in melanocytes. Created in https://BioRender.com

Here, we presented the multimodal laser strategy, employing a 1550 nm fractional laser to target tissue texture and collagen induction with the addition of a 577 nm yellow laser to reduce erythema and vascular hyperreactivity [15,16]. Following the laser therapy, the UCMSC-exosomes were incorporated as a key component of the treatment strategy to modulate the scar healing environment (Figure 2). The UCMSC-exosomes (BIONET Therapeutics, Taiwan) contain bioactive molecules, including signaling proteins and microRNAs, which modulate inflammatory responses and promote tissue repair. For example, similar to existing literature [17], we found the UCMSC-exosomes contain NT5E/CD73 and miR-21 based on the proteomic and miRNA microarray data, potentially modulating the inflammatory signaling, such as NF-kB and IL-6/JAK/STAT signaling, stimulated from the laser therapy (data not shown). Additionally, multiple growth factors, such as FGF, HGF, TGF-β, and even TGF-β antagonist Decorin, were found in the UCMSC-exosomes, necessitating a controlled wound healing process (data not shown). The cross-interactions among these proteins suggest a delicate modulation of cell proliferation, collagen synthesis and organization, and an increase of angiogenesis, all central to the pathophysiology of hypertrophic scarring [18]. Based on the results, we hypothesized that the observed early reduction in pruritus, pain, and erythema after the first treatment session, and the observed progressive improvement in scar texture and smoothness after the second session, reflect the enhancement of extracellular matrix remodeling and fibroblast modulation likely mediated by UCMSC-exosomes, potentially augmented by microneedling-induced microchannels that facilitate dermal delivery of exosomes during scar maturation [19,20].

Several limitations of this report must be acknowledged. First, as a single case report, the findings cannot be generalized or used to establish a definitive treatment protocol. Second, the absence of a control group precludes attribution of clinical improvement to any single component of the multimodal protocol, and the independent contribution of UCMSC-exosomes as the primary driver of improvement cannot be confirmed. Third, objective quantitative assessment tools - including dermoscopy, colorimetry, and 3D surface imaging - were not available in this clinical setting; future studies should incorporate these modalities alongside validated symptom scales such as the Visual Analogue Scale (VAS) for pain and a standardized pruritus scale. Fourth, no direct biomarker validation was performed to confirm the in vivo activity of the exosome cargo. Finally, the mechanistic discussion presented herein, including the proposed roles of NT5E/CD73, miR-21, and associated growth factors, is hypothetical and based on existing literature rather than direct experimental evidence from this case. These limitations underscore the need for prospective, randomized, controlled trials to validate this multimodal approach.

## Conclusions

This case report suggests the potential efficacy of a combined treatment dual-wavelength fractional laser protocol (1550 nm + 577 nm) in combination with UCMSC-derived exosomes. The integration of exosome-based therapy with laser represents a promising, multimodal approach that may leverage both biologically active signaling and energy-based tissue modulation. Clinical observation revealed no adverse events throughout the treatment course, with a possible anti-inflammatory protective effect suggested by the absence of PIH. Nevertheless, as the clinical application of exosomes remains in the early phase, further prospective, randomized, controlled research and comprehensive mechanistic data are essential to establish a robust and validated exosome treatment protocol for future dermatological applications.
